# Neuroimaging Correlates of Alzheimer’s Disease biomarkers in a mid‐life, racially diverse high‐risk cohort

**DOI:** 10.1002/alz.092741

**Published:** 2025-01-09

**Authors:** Maria Misiura, Chinkuli Munkombwe, Kay C. Igwe, Danielle D Verble, Kelly D. S. Likos, Henrik Zetterberg, Vonetta M Dotson, Adam M. Brickman, Whitney Wharton

**Affiliations:** ^1^ Georgia State University, Atlanta, GA USA; ^2^ Columbia University, New York, NY USA; ^3^ Emory University, Atlanta, GA USA; ^4^ Institute of Neuroscience and Physiology, Sahlgrenska Academy at the University of Gothenburg, Gothenburg Sweden; ^5^ University of Gothenburg, Mölndal, Gothenburg Sweden; ^6^ Department of Psychiatry and Neurochemistry, Institute of Neuroscience and Physiology, The Sahlgrenska Academy, University of Gothenburg, Mölndal, Gothenburg Sweden; ^7^ UK Dementia Research Institute at UCL, London UK; ^8^ Taub Institute for Research on Alzheimer's Disease and the Aging Brain, New York, NY USA; ^9^ The Gertrude H. Sergievsky Center, College of Physicians and Surgeons, Columbia University, New York, NY USA; ^10^ Department of Neurology, Vagelos College of Physicians and Surgeons, Columbia University, and the New York Presbyterian Hospital, New York, NY USA

## Abstract

**Background:**

Clinicians and researchers utilize neuroimaging (NI) biomarkers of Alzheimer’s disease (AD) at an increasing rate. It is crucial that we determine whether these biomarkers generalize to underrepresented populations, particularly Black Americans (BAs), as they are 64% more likely as white individuals to develop AD. BAs may exhibit unique AD biomarker profiles across disease states, including NI biomarkers. Investigating biomarkers in at‐risk individuals may allow for early identification and intervention. Here we analyze comprehensive NI biomarkers including resting state fMRI, white matter lesions, and hippocampal volumes in conjunction with cerebrospinal fluid (CSF) markers in a midlife, racially diverse, cognitively normal cohort with a parental history of AD.

**Method:**

Data included 57 cognitively normal, middle‐aged individuals, 14 BAs and 43 white. CSF was acquired via lumbar puncture to determine CSF t‐tau and Aβ_1–42_ concentrations. Using resting state fMRI data, we analyzed default mode network connectivity between the posterior cingulate & precuneus, parrahippocampal gyrus (PHG), temporal pole, hippocampus, and lateral temporal cortex. We obtained regional WMH data from FLAIR images using an in‐house algorithm, and hippocampal volumes from Freesurfer. In our multivariate model, outcome variables were connectivity values, regional WMH and hippocampal volumes, with race and CSF t‐tau and Aβ_1–42_ as our independent variables with a race X CSF interaction term.

**Result:**

We identified a significant race X t‐tau and race X Aβ_1–42_ interaction term for temporal and parietal WMH volumes and connectivity between PHG and temporal pole. Higher AD CSF biomarkers negatively correlated with brain connectivity (p=0.01), and positively with WMH volume (p<0.001) in BAs.

**Conclusion:**

We extend our previous work to a middle‐aged cohort to show that DMN connectivity may be a predictor of AD risk in middle‐age, particularly for BAs, and that BAs may exhibit earlier vulnerability to vascular lesions. We support previous work that the temporal lobe is the first region to experience AD‐related connectivity changes, and that WMHs may predispose this region to AD vulnerability. This study emphasizes the need for recruitment of diverse cohorts in NI studies of AD.

